# Automated CT-based visceral fat density predicts mortality regardless of visceral fat area

**DOI:** 10.1093/bjr/tqag001

**Published:** 2026-01-12

**Authors:** Adam J Kuchnia, Glen M Blake, Matthew H Lee, Jevin Lortie, John W Garrett, Perry J Pickhardt

**Affiliations:** Department of Nutritional Sciences, University of Wisconsin, Madison, WI 53706, United States; School of Biomedical Engineering and Imaging Sciences, King’s College London, St. Thomas’ Hospital, London SE1 7EH, United Kingdom; Department of Radiology, University of Wisconsin School of Medicine and Public Health, Madison, WI 53792-3252, United States; Department of Nutritional Sciences, University of Wisconsin, Madison, WI 53706, United States; Department of Radiology, University of Wisconsin School of Medicine and Public Health, Madison, WI 53792-3252, United States; Department of Radiology, University of Wisconsin School of Medicine and Public Health, Madison, WI 53792-3252, United States

**Keywords:** automated AI-based CT, visceral adipose tissue (VAT), Hounsfield units (HU), VAT area, VAT HU, mortality

## Abstract

**Objectives:**

We evaluated whether automated CT-based adiposity tools can predict all-cause mortality in a large retrospective adult population.

**Methods:**

This study included 151 177 patients who underwent abdominal CT between 2000 and 2021. An AI-based algorithm measured abdominal visceral adipose tissue (VAT) cross-sectional area and density at the L3. Kaplan–Meier survival curves and hazard ratios assessed VAT and mortality.

**Results:**

Among 136 895 patients included, 9059 died within 1 year and 18 829 died within 2 to 20 years post-CT. Higher VAT density predicted 1-year mortality (hazard ratio [HR] up to 3.8) and over 2-20 years (HR up to 2.1). In contrast, VAT area did not significantly predict mortality. High VAT density was associated with the poorest survival, regardless of area. Low VAT density predicted better survival, regardless of area. VAT density consistently predicted mortality across age groups and sexes, whereas BMI did not differentiate risk.

**Conclusions:**

AI-enabled CT measures of VAT density are superior to VAT area for predicting all-cause mortality. Furthermore, we analysed VAT density vs. BMI in our largest age group (40-59) and found BMI was unable to adequately predict risk of mortality. Automated assessment of VAT density may enhance patient risk assessment and management.

**Advances in knowledge:**

Assessing visceral fat density using fully automated AI-based CT tools offers a significant advancement in predicting health risk, leading to targeted interventions and improved management strategies. This study is novel due to its large patient population, offering evidence that prognostication with VAT density is broadly generalizable across varying patient populations.

## Introduction

In the United States, it is expected that the 65 and over population will nearly double by 2050.^1^ Over the same time, obesity is expected to surpass its current level of 42%, substantially increasing the risk for type 2 diabetes, cardiovascular disease, some cancers, Alzheimer’s, and all-cause mortality.^2^ Common approaches used to evaluate obesity, including body mass index (BMI) and waist circumference, do not fully address the disease risk associated with visceral adiposity, thereby providing an incomplete assessment of cardiometabolic health.^2^^,^^3^ Conversely, fully automated artificial intelligence (AI) CT tools can derive objective body composition measures,^4^ including the separate discrimination of subcutaneous adipose tissue (SAT) from visceral adipose tissue (VAT).^5^ This provides an improved assessment of cardiometabolic risk as visceral fat is strongly associated with consequential cardiovascular events and is an independent predictor of all-cause mortality.^6^^,^^7^ Several studies have separately analysed CT-derived SAT and VAT quantity; however, few have examined characteristics beyond VAT quantity when considering risk of death in a large population of adults.^6^^,^^8^

VAT quantity increases with age and has important consequences for predicting disease^9–11^; however, morphological changes and infiltration of varying cell types within adipose tissue may be more important to maintaining proper adipocyte health and function. Such changes include hypertrophy, hypoxia, fibrosis, and immune cell infiltration,^12^^,^^13^ which have been shown to dictate CT-measured adipose tissue density.^14^ Fully automated CT methods outperform standard clinical tools for predicting risk in healthy screening cohorts^15^ and can be used to capture morphological changes that associate with increased adipose tissue density. The objective of this study was to assess the utility of fully automated CT-based VAT quantity and VAT density for predicting all-cause mortality in a large cohort of adults. The hypothesis that VAT density improves the prediction of mortality over VAT quantity will be tested.

## Methods

### Patient cohort

**Figure 1.**
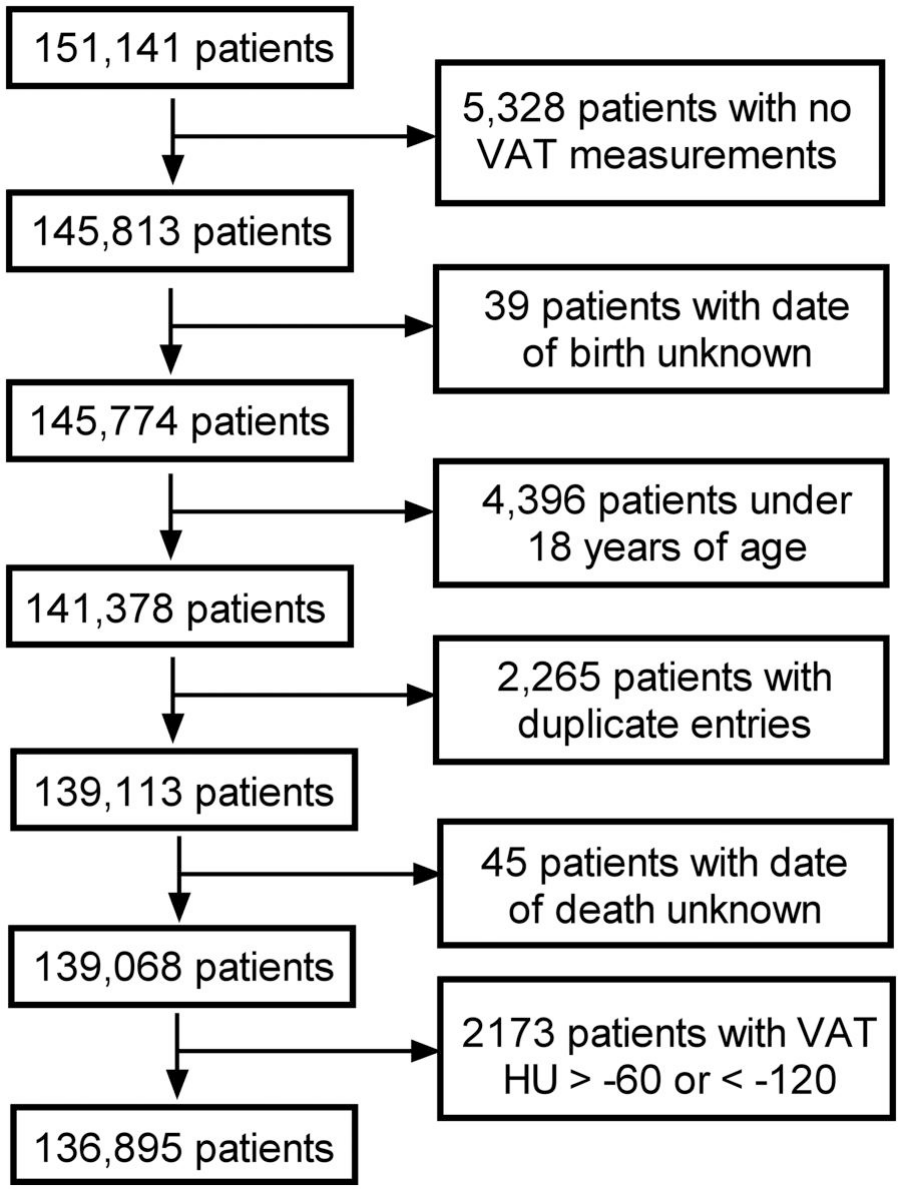
Flowchart explaining the factors for excluding subjects from the final study dataset.

This retrospective Health Insurance and Portability and Accountability Act-compliant cross-sectional investigation was approved by the Institutional Review Board. The requirement for informed consent was waived. Fully automated AI-based algorithms for quantifying abdominal VAT were applied to abdominal CT scans in 151 141 patients undergoing a CT examination between January 2000 and March 2021. Patients were excluded for AI tool failure due to missing analysis or extreme outlier values, younger than 18 years of age, errors of identical duplicate entries, and errors in reporting of patient death (Figure 1). To clarify omission of extreme outlier values from AI tool, VAT density values above −60 HU and below −120 HU were omitted due to reliability concerns of the AI tool when VAT area becomes extremely small, including non-VAT tissue in the analysis or being affected by partial-volume effects leads to erroneous measurements. For 5328 patients, VAT measurements were not able to be obtained (Figure 1), likely due to scans that are either scouts, either superior or inferior to the L3, or corrupted files.

### CT technique

Patients underwent assorted CT protocols that included the abdomen. These protocols included various contrast phases, X-ray tube potentials, and dose ranges. Many different scanners from various manufacturers were included.

### Fully automated artificial intelligence CT fat biomarkers

Each DICOM dataset underwent pre-processing includes several key steps: (1) Starting with the DICOM files of a given series, ensure patient orientation is standard left-anterior-superior (LAS) voxel ordering, (2) Volume slice sampling equalization and resampling to uniform 3 × 3 mm sampling and thickness, (3) CT number normalization (removing vendor Hounsfield unit [HU] offset), and (4) Conversion to single NIFTI file per series for processing. All 4 data preparation and normalization steps are performed automatically using a program implemented as a mix of Bash and Python scripts. The next step was the localization of the first and third lumbar vertebral bodies (L1 and L3 levels). These levels are used to ensure adequate coverage of the abdomen as well as to localize the fat measurements to a consistent anatomical position. The localization of the vertebral bodies is automatically performed using a convolutional neural network (CNN)-based unsupervised body part regression (UBR) algorithm.^16^^,^^17^ This deep learning model is implemented in Caffe^18^ and provides predicted levels for the twelfth thoracic (T12) through the fourth lumbar (L4) vertebral bodies as slice numbers.

An automated segmentation of abdominal fat at the L3 level was then performed.^5^ This semantic segmentation classifies all voxels as either visceral fat, subcutaneous fat, or neither; this is achieved using fuzzy c-means to cluster fatty tissues and active contour models to separate subcutaneous and visceral fat.^19^ From this segmented image, the areas of SAT, VAT, and total adipose tissue (TAT) are calculated (in mm^2^) as well as the CT HU of the visceral fat compartment.

Intravenous contrast is an important factor to consider, as it can affect measured tissue density.^20–22^ VAT was calculated from abdominal CT scans across various contrast phases. When multiple scans were available for a patient, the AI tool automatically selected the scan using the following order of preference: non-contrast first, followed by portal venous phase, while avoiding arterial phase when possible. In our full dataset of 151 141 patients, 62.7% of scans were contrast-enhanced, and 37.3% were non-contrast. Contrast correction was applied to all contrast-enhanced scans as follows: VAT area values were adjusted by a factor of 1.063 (independent of area), and VAT HU values were corrected by adding +3.9 HU at −120 HU, increasing linearly to +6.8 HU at −60 HU. The 0.03% of scans with unknown contrast status were not corrected for enhancement.^23^

### Clinical outcomes measures

Clinical variables including patient age, sex, and BMI were obtained from the electronic health record (EHR). The outcome of interest following CT was death of any cause. The date of the event (ie, death) was determined from the EHR. For patients who did not have an event, the date of the last recorded clinical follow-up in the EHR was documented.

### Statistical analysis

Men and women (self-reported) were analysed independently, and data were pooled if differences between their survival indices were too small to be clinically significant. To minimize the effect of age on survival indices, patients were divided into 4 age groups (18-39, 40-59, 60-79, and 80+ years). Dates of deaths and dates of last contact were used to generate Kaplan–Meier (KM) survival curves. For each sex and each age group, patients were divided into 4 quartiles (Q1 to Q4) according to their VAT area. Each quartile was further subdivided into those with VAT HU values above or below the median value. Patients in Q1 or Q2 with VAT HU values below their respective medians were assigned to the “Low Area/Low HU” group. Patients in Q1 or Q2 with HU values above their medians were assigned to the “Low Area/High HU” group. Patients in Q3 and Q4 were similarly assigned to the “High Area/Low HU” and “High Area/High HU” groups, as appropriate. KM survival curves were generated for each of these 4 VAT group combinations for each age group, and hazard ratios were calculated using the log-rank test. Hazard ratios were also plotted against VAT HU in intervals of 5 HU after pooling all VAT area data. Unlike VAT HU, measurements of VAT area varied strongly with sex and age. To control for this, HR ratios were plotted in percentile bins allowing for sex and age group. For men and women in the 40- to 59-year age group with a body mass index (BMI) measurement within 1 year of their CT scan, KM survival curves were created according to their BMI quartile and compared directly with the KM curves for the 4 VAT area/HU groups in the same subjects. Statistical analysis was performed using Statistics Kingdom software (Statistics Kingdom, Melbourne, Australia). A *P* value <.05 was considered statistically significant. The 95% confidence intervals were computed using Poisson statistics.

## Results

### Patient sample

The final study sample consisted of 136 895 adults (mean age, 53 years; 52% female) who underwent abdominal CT imaging for any reason, as outlined in Figure 1. Participants were predominantly White at 89.1%, with additional representation from Black 5.1%, Asian 2.2%, American Indian 1.1%, Unknown or no information 1.5%, declined to answer 0.8%, and Hawaiian 0.2%. A total of 9059 patients died in the first year (year 1) following the CT exam and 18 829 died in 2 to 20 years (years 2-20) following the CT exam. At the 20-year point, the mean follow-up time was 6.4 years, the median follow-up time was 4.9 years, 27 888 (20.4%) patients had died, 105 127 (76.7%) were lost to follow-up, and 3880 (2.8%) remained in the study.

### Fat-based body composition measures

**Figure 2.**
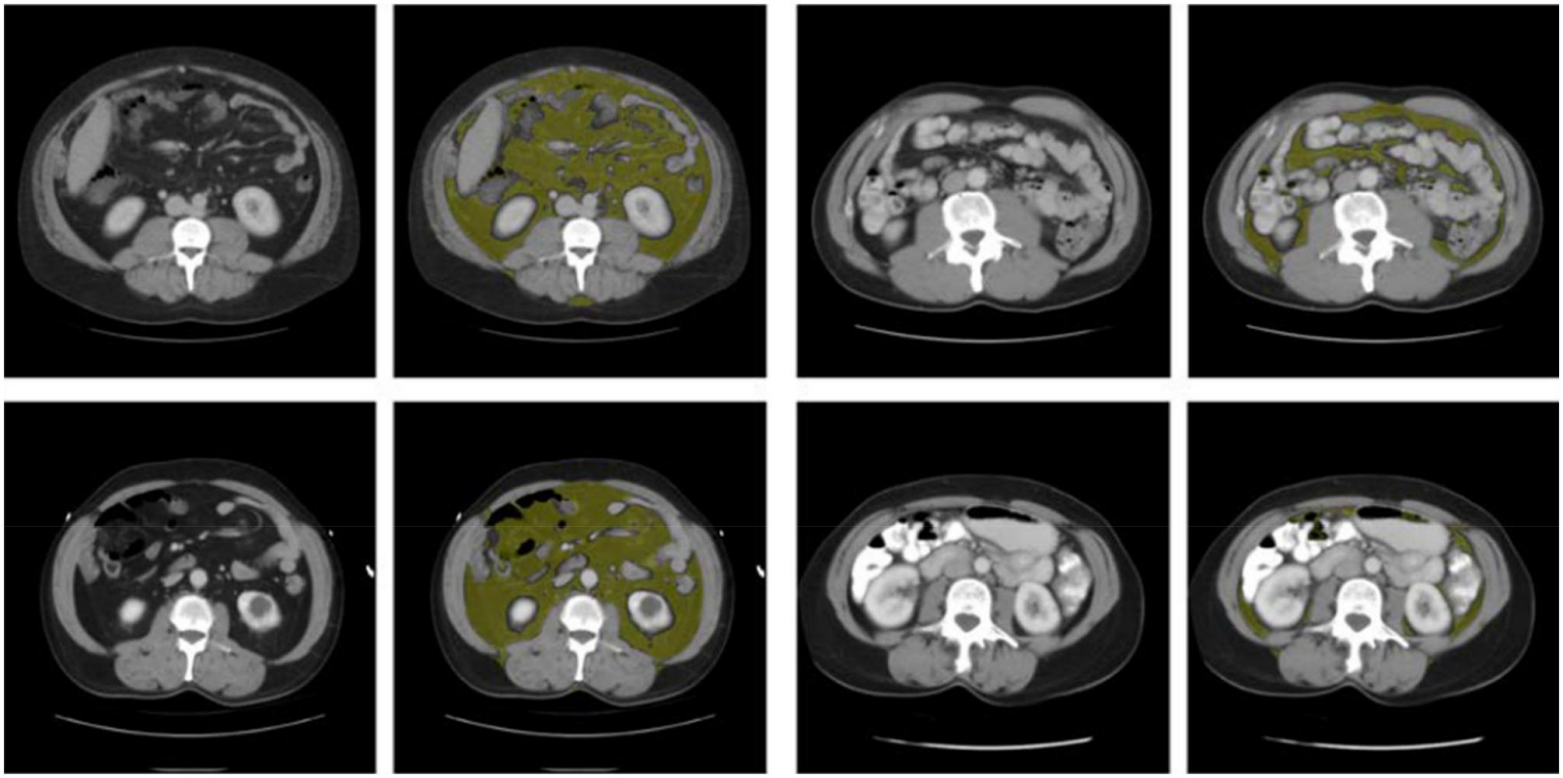
Axial CT images without (left) and with (right) depiction of the automated segmentation of visceral adipose tissue (VAT) at the L3 level in 4 patients, corresponding to high VAT area/high VAT HU (upper left), high VAT area/low VAT HU (lower left), low VAT area/high VAT HU (upper right), and low VAT area/low VAT HU (lower right) combinations.

**Figure 3.**
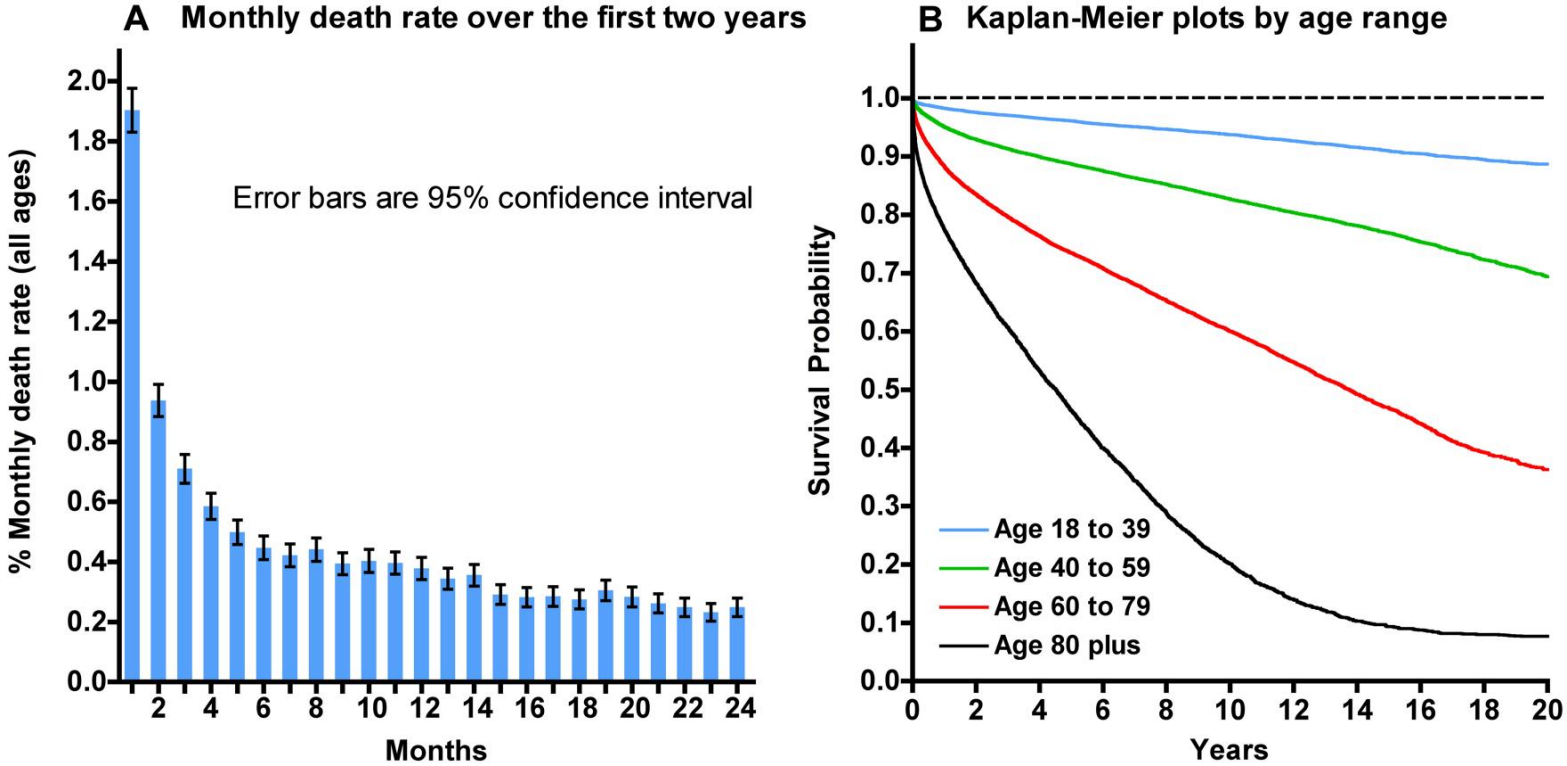
(A) Monthly death rate of study subjects during the first 2 years following the date of the CT scan. The monthly death rate is plotted as a percentage of the number subjects still alive and not lost to follow-up. The error bars were calculated using Poisson statistics. (B) Kaplan–Meier (KM) survival curves for the first 20 years of the study for subjects in 4 age ranges (ages at baseline: 18-39, 40-59, 60-79, and 80+ years).

Patient characteristics by age and fat body composition measures are summarized in Table 1. The largest age group was the 40-59 years (*n* = 52 282). In general, mean VAT area increased with age until age 60-79 years, thereafter decreasing in adults 80 years of age and older in both men and women, going from 76 cm^2^ to 128 cm^2^ to 154 cm^2^, before dropping to 136 cm^2^ in women (*P < .*0001), and in men, going from 121 cm^2^ to 215 cm^2^ to 259 cm^2^, before dropping to 247 cm^2^ (*P < .*0001). Mean VAT density measured in HU showed no such trend with ageing. Patient characteristics by age and VAT area/density combinations are summarized in Table S1 (see online supplementary material). Figure 2 shows representative examples of abdominal VAT measures for each of the 4 combinations according to area and density. Figure 3 shows monthly deaths over the first 2 years following the CT exam and Kaplan–Meier plots of survival probability up to 20 years post-CT exam by age range. As expected, age is a strong predictor of overall survival with 80 years of age and older driving increased deaths in the first 2 years post-CT, followed by 60-79 years of age.

**Table 1.** Patient characteristics.

	Men					Women				
Age group (y)	*N*	Mean age (SD)	Mean BMI (SD)^a^	Mean VAT area (SD) (cm^2^)	Mean VAT HU (SD)	*N*	Mean age (SD)	Mean BMI (SD)^a^	Mean VAT area (SD) (cm^2^)	Mean VAT HU (SD)
All ages	65 596	53.3 (17.3)	29.1 (6.4)	210.1 (137.7)	−90.7 (10.1)	71 299	52.3 (17.7)	29.4 (8.2)	123.2 (102.8)	−88.1 (9.5)
Age18-39	15 318	29.2 (6.3)	27.9 (6.6)	121.4 (102.5)	−88.8 (9.7)	18 262	29.1 (6.3)	28.8 (8.5)	76.1 (78.3)	−85.7 (8.7)
Age 40-59	24 694	50.6 (5.6)	29.9 (6.7)	216.0 (130.0)	−91.8 (10.0)	27 588	50.2 (5.6)	30.1 (8.5)	128.2 (103.4)	−89.2 (9.4)
Age 60-79	21 723	68.0 (5.5)	29.3 (6.0)	259.4 (139.2)	−91.1 (10.0)	20 890	68.1 (5.6)	29.6 (7.9)	154.8 (108.5)	−89.3 (9.7)
Age 80 plus	3861	84.4 (3.8)	27.3 (4.7)	247.9 (129.4)	−88.7 (10.5)	4559	85.2 (4.2)	26.6 (5.8)	136.3 (90.7)	−85.9 (10.1)

Abbreviations: BMI = body mass index; HU = Hounsfield Units; SD = standard deviation; VAT = visceral adipose tissue.

a Men (72%) and 74% of women with VAT area and HU measurements had a BMI measurement within 365 days of the date of their CT scan.

**Figure 4.**
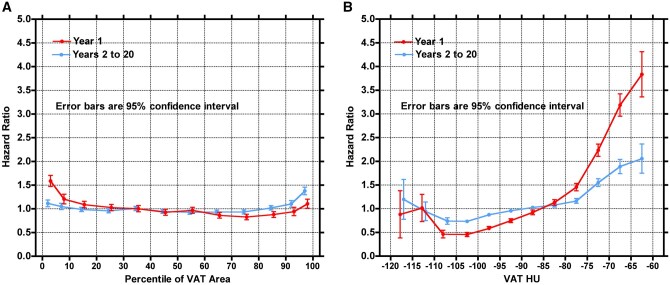
(A) Relative risk of death as a function of the percentile of VAT area. Percentiles were calculated separately for men and women and each age group and pooled for this figure. The VAT areas were binned in the following percentiles: 0%-5%, 5%-10%, 10%-20%, 20%-30%, ……, 80%-90%, 90%-95%, and 95%-100%. Curves for the first year and for years 2 to 20 are plotted separately. Data were pooled for both sexes and all age groups. Error bars are ±1.96 SE. (B) Relative risk of death as a function of VAT HU in intervals of 5 HU from −120 to −60. Curves for the first year and for years 2 to 20 are plotted separately. Data were pooled for both sexes and all 4 age groups. Error bars are ±1.96 SE.

Relative risk of death as a function of VAT area and VAT density for both post-CT year 1 and years 2 to 20 are shown in Figure 4. Increased VAT density increased risk of death starting just above −85 HU. The HR steadily increased after −100 HU for both time periods, with lowest risk of death occurring just below −100 HU and the highest just above −65 HU. At their peak, the HR approached 4.0 in year 1 and 2.0 in years 2-20. Conversely, VAT area did not confer increased risk, with a HR hovering around 1.0, excluding extreme low and high ends, where risk of death slightly increased. Justification to fit a single curve for all age groups is shown in Figure S1 (see online supplementary material for a color version of this figure), highlighting similar trends in all age groups.

**Figure 5.**
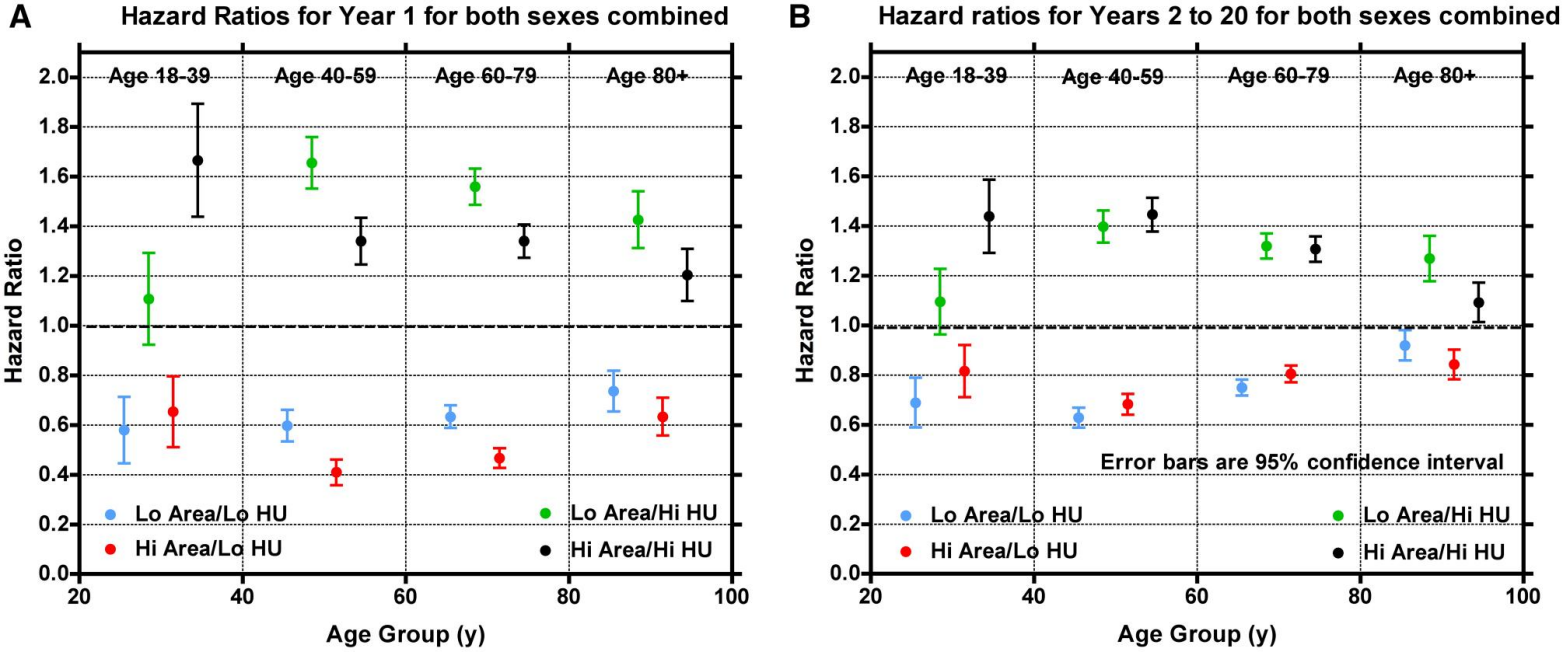
(A) Relative risk of death in the first year of the study as a function of the VAT area and VAT HU group subjects were assigned to. Subjects in the Lo Area groups have a VAT area below the median for their sex and age group. Subjects in the Hi Area groups have a VAT area above the median for their sex and age group. Subjects in the Lo HU groups have a VAT HU below the median for their VAT area quartile. Subjects in the Hi HU groups have a VAT HU above the median for their VAT area quartile. Hazard ratios were calculated separately for each of the 4 age groups in the study. Error bars are ±1.96 SE. (B) Same as (A), except showing the risk of death in years 2 to 20.

Table S2 (see online supplementary material) lists the thresholds for VAT area quartiles and corresponding median VAT density thresholds created to evaluate the relative risks between the 4 VAT groups (ie, Low/Low, High/High, High/Low, and Low/High for VAT area/HU, Low = below median, High = above median). Figure 5 and Table 2 show the results of the mortality time-to-event analysis for each of these 4 VAT groups. The highest risk of death in both year 1 and years 2-20 were driven by Hi VAT density, regardless of VAT area. VAT density similarly predicted risk in all age groups and increased risk of death persisted regardless of VAT area. The highest year 1 risk by age group was the combination of high VAT area and high VAT density (HR = 1.67, *P < .*001) in years 18-39, followed by low VAT area and high VAT density in years 40-59, 60-79, and over 80 years (HR = 1.66, 1.56, 1.43, respectively; *P < .*001). Figure S2 (see online supplementary material for a color version of this figure) showed that the risks in men and women were similar when looking at years 40-59 and justified grouping sexes for Figures 5 and Table 2.

**Table 2.** Hazard ratios and 95% confidence intervals for the different VAT groups.

		Men and women year 1		Men and women years 2 to 20		*P* value year 1 vs. years 2 to 20
Age group (y)	VAT group	Hazard ratio (95% CI)	*P* value of hazard ratio	Hazard ratio (95% CI)	*P* value of hazard ratio	
Age 18-39	Low area/low HU	0.579 (0.134)	***P* < .001**	0.689 (0.100)	***P* < .001**	*P* = .194
	Low area/high HU	1.107 (0.185)	*P* = .256	1.095 (0.132)	*P* = .159	*P* = .913
	High area/low HU	0.653 (0.142)	***P* < .001**	0.816 (0.105)	***P* < .001**	*P* = .070
	High area/high HU	1.665 (0.227)	***P* < .001**	1.439 (0.148)	***P < .*001**	*P* = .102
Age 40-59	Low area/low HU	0.597 (0.063)	***P* < .001**	0.628 (0.040)	***P < .*001**	*P* = .408
	Low area/high HU	1.655 (0.104)	***P* < .001**	1.397 (0.065)	***P < .*001**	***P* < .001**
	High area/low HU	0.409 (0.052)	***P* < .001**	0.682 (0.042)	***P* < .001**	***P* < .001**
	High area/high HU	1.340 (0.094)	***P* < .001**	1.446 (0.068)	***P* < .001**	*P* = .073
Age 60-79	Low area/low HU	0.633 (0.046)	***P* < .001**	0.749 (0.032)	***P* < .001**	***P* < .001**
	Low area/high HU	1.559 (0.073)	***P* < .001**	1.319 (0.051)	***P* < .001**	***P* < .001**
	High area/low HU	0.466 (0.040)	***P* < .001**	0.804 (0.034)	***P* < .001**	***P* < .001**
	High area/high HU	1.340 (0.067)	***P* < .001**	1.307 (0.051)	***P* < .001**	*P* = .445
Age 80-109	Low area/low HU	0.736 (0.082)	***P* < .001**	0.919 (0.061)	***P* = .009**	***P* < .001**
	Low area/high HU	1.426 (0.114)	***P* < .001**	1.269 (0.091)	***P* < .001**	***P* = .036**
	High area/low HU	0.633 (0.076)	***P* < .001**	0.842 (0.060)	***P* < .001**	***P* < .001**
	High area/high HU	1.204 (0.105)	***P* < .001**	1.092 (0.079)	***P* = .022**	*P* = .096

Bold characters denote statistical significance

**Figure 6.**
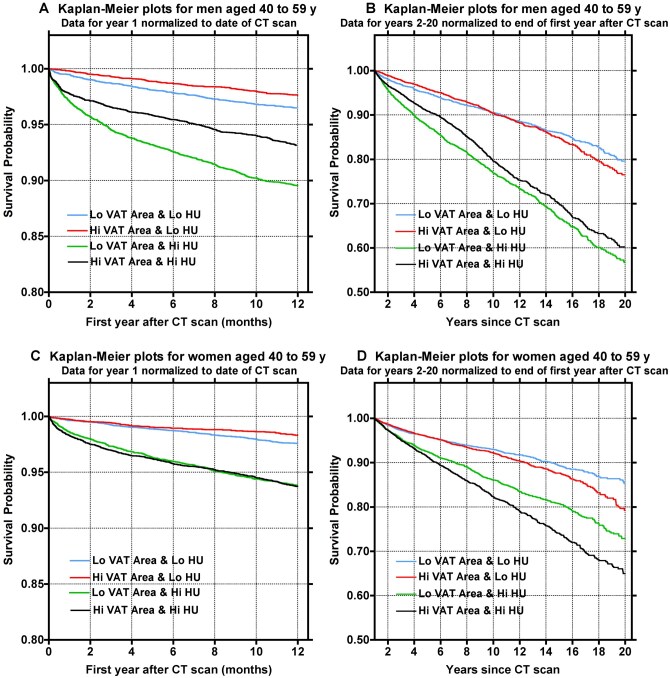
(A) Kaplan–Meier (KM) survival curves for the first year of the study for men aged 40 to 59 years at the time of their CT scan. KM curves are plotted separately according to the VAT area and VAT HU group that subjects were assigned to (ie, Lo Area and Lo HU; Hi Area and Lo HU; Lo Area and Hi HU; Hi Area and Hi HU). The definitions of these groups are explained in the legend to Figure 5. (B) Same as (A), except that the KM curves are plotted for the deaths of men between the end of Year 1 and the end of Year 20 since the date of their CT scan; (C) KM curves for the first year of the study for women aged 40 to 59 years at the time of their CT scan. (D) Same as (C), except that the KM curves show the deaths of women between the end of Year 1 and the end of Year 20 since the date of their CT scan.

Kaplan–Meier survival probability plots assessing the same combinations of VAT area and VAT density in the largest age 40-59 years subset are shown in Figure 6. The worst survival was observed in both men and women with high VAT density and either high VAT area (black curve) or low VAT area (green curve) in both year 1 and years 2 to 20. Patients with high VAT density (green or black curves) are visually distinguishable from those with low VAT density (red or blue curves), implying increased risk associated with high VAT density, regardless of VAT area. Upon inspection of Kaplan–Meier survival probability plots assessing quartiles of BMI, there is relatively little visual separation of any BMI quartile or difference in mortality time-to-event between quartiles compared with the similar plots for the 4 VAT groups (Figure S3—see online supplementary material for a color version of this figure).

Natural logarithm plots of the hazard ratios for VAT density and VAT area percentile show each as a burden on ageing (Figure S4—see online supplementary material for a color version of this figure). For those patients who died within year 1 of their respective CT exam, a 10% increase in percentile of VAT area is equivalent to being 9 months younger, whereas a 10 HU increase in VAT is equivalent to 11 years of ageing. Upon inspection of those having died in the 2 to 20 years following CT, a 10% increase in percentile of VAT area is equivalent to being 2 weeks younger and is not statistically significant; however, a 10 HU increase in VAT is equivalent to 4 years of ageing.

## Discussion

In this study, fully automated CT-based measures of visceral fat were assessed in 136 895 adults having an abdominal CT exam. VAT density was a strong, significant predictor of death in the first year following the CT exam and also throughout the period from year 2 to year 20. Conversely, VAT area was not a significant predictor of death. These results were similar between sexes and in all age ranges. Combinations of VAT density and VAT area showed high VAT density as the driver of risk, regardless of having high or low VAT area. As such, of the 4 different combinations (ie, Low Area/Low HU, High Area/Low HU, Low Area/High HU, and High Area/High HU), the lowest risk was in both groups having low density, and the highest risk was in both groups having high density. These results persisted in both sexes and in all age groups. In contrast, BMI was unable to adequately predict the risk of death (only assessed in our largest age group, 40-59 years old). These findings highlight the role of VAT density in predicting survival and can be derived using a fully automated AI CT fat tool.

### Risk associated with visceral fat quantity in ageing

Obesity is a well-established risk factor for all-cause mortality; however, ageing is associated with a redistribution of adipose tissue in the abdominal region that may better predict risk. This redistribution and ectopic accumulation of lipids is marked by metabolic dysregulation within subcutaneous adipocytes, resulting in the inability of subcutaneous fat to take up excessive circulating free fatty acids.^24^^,^^25^ The shunting of lipids away from subcutaneous fat and increasing uptake within VAT increases with age and is a stronger predictor of consequential outcomes than total adiposity or BMI.^8^^,^^26^ Although the present results do show an increase in VAT area with age, it is a weak predictor of death after controlling for age. Although risk is slightly increased in the 2 age groups <60 years old, it is largely absent in those ≥60 years of age (data not shown). This may be partly explained by a partial reversing of VAT area in individuals in the latter stages of life. These results largely confirm results from a large systematic review and inspection of risk associated with VAT quantity in several cohorts of predominantly healthy adults.^8^ These data may speak to the importance of other clinical variables, including alcohol consumption, smoking, and physical activity that may eclipse the consequences of VAT area and challenge the long-held concept that VAT area is inherently associated with poor outcomes regardless of age. Less is known about additional automated CT-based VAT measures and their long-term consequences in relation to VAT area.

### Risk associated with visceral fat density in ageing

The consequences of high VAT density found here may be the result of age-related white adipose tissue dysfunction, leading to insulin resistance, inflammation, and obesity.^12^^,^^13^ In the setting of increased white adipose tissue hypertrophy and expansion, a remodelling of adipose tissue occurs due to a reduction in vascularity and oxygen availability with subsequent upregulation of genes that produce collagens.^27^ This has been demonstrated in VAT of older mice, highlighting a dramatic increase in collagen staining resulting in fibrosis.^28^ Hypertrophic growth associated with fibrosis is a key factor driving adipose tissue dysfunction in ageing^12^ and can be measured using CT-based methods. These age-related cellular and molecular changes that occur within adipose tissue may provide context to the consequential effects of increasing VAT density found in this study.

Within the Health ABC Study (*n* = 898), VAT density was associated with mortality even after adjusting for total fat and BMI in healthy adults with a mean age of 73 years.^29^ Similar results were shown within a younger, healthy adult population, approximately 50 years of age, in the Framingham Heart Study (*n* = 3324), where VAT density was associated with all-cause and cancer mortality.^30^ These results likely point to the consequential effects of metabolic dysfunction that accompanies hypertrophic growth of white adipose tissue in ageing. Our results extend upon these studies and show that a high VAT density is associated with an increased risk of death at all ages from 18 to greater than 80 years old.

### Risk associated with visceral fat density in disease

White adipose tissue dysfunction results in morphological changes induced by disease-associated inflammation, fibrosis, and immune cell infiltration. CT-based adipose tissue density may be similarly reflective of these metabolic abnormalities. In patients with liver disease presenting with cirrhosis, SAT density was associated with increased mortality.^14^ The increase in SAT density was confirmed with biopsy, illustrating histological features of adipocyte atrophy, expanded extracellular matrix, macrophage infiltration, and subsequent fibrosis.^14^ CT-measured SAT and VAT density are similarly associated with overall survival in patients with pancreatic cancer.^31^ This may be explained by the hypermetabolic response to cancer, causing a systemic inflammatory response, immune cell infiltration, and white adipose browning through upregulation of uncoupling protein 1 (UCP1).^32^ The induction of UCP1 is accompanied by the histological attributes of smaller, atrophic adipocytes with expanded fibrotic extracellular matrix. The prognostic ability of similar radiological features of VAT and SAT density has also been assessed in those affected by sarcoma; however, results are conflicting.^33^^,^^34^

The creation of AI-based body composition tools offers insight into characteristics of abdominal fat that may better predict consequential outcomes. These rapid and reproducible tools not only have the ability to transform clinical care by personalizing medical practice but also predict unique risk profiles attributed to large communities and populations. We do, however, acknowledge some limitations to this study. First, this is a large retrospective cohort of individuals obtaining a CT exam for any indication; some will be healthy routine screening cohorts, while others will be for the identification and staging of fulminant disease processes. Due to the size of our population and limitations with consistencies in medical record data, it was unfeasible to differentiate between healthy participants and those with disease. In addition, we found our AI tool was unreliable at extremely low VAT area measurements, leading to excluded participants. It is our hope that this will be improved with future iterations of the tool that will have additional training on low-VAT images. Due to the large size of our cohort, it was not feasible to manually correct for these issues. Relatedly, it also was not feasible to assess for image problems that would have affected measurements, such as clinical abnormalities such as ascites or anasarca, or artefact abnormalities, such as those caused by implanted hardware. The authors acknowledge that mortality data from the National Death Index (NDI) may be more complete than obtaining mortality data from the health record. However, with the large sample size, linking participants to the NDI was operationally intensive and unfeasible for this study. Finally, many studies use VAT index as a metric. However, the scale of our cohort prevented us from obtaining consistent height data on all subjects, therefore we report unadjusted VAT HU values.

Regardless, CT radiological assessment can identify adipose tissue abnormalities due to ageing and disease. Both scenarios cause consequential metabolic dysfunction, leading to poor outcomes that can be detected using automated CT-based tools. Second, this large cohort is a mix of both contrast and non-contrast exams, although we have applied previously established correction factors, resulting in non-contrast equivalent results for contrast-enhanced exams.^23^ Regardless, because adipose tissue is less perfused than skeletal muscle, the effect of contrast may be negligible.^35^ Lastly, the patient cohort analysed was predominantly white, precluding further analysis by race or ethnicity. This should be addressed upon follow-up by more diverse multi-centre cohorts.

In conclusion, fully automated AI-based CT body composition tools to derive VAT measures can be utilized to assess risk in large cohorts. High VAT density is an important predictor of mortality risk regardless of VAT area. These findings highlight the importance of morphological attributes of VAT that increase its density rather than VAT quantity. Clinical implementation of these tools can enhance patient-centred care through a more comprehensive approach to mortality risk prediction using data that currently go unutilized.

## Supplementary Material

tqag001_Supplementary_Data

## Data Availability

Data will be made available on reasonable request to the corresponding author.
