# Supplementary material for: Automated CT-based visceral fat density predicts mortality regardless of visceral fat area
Source: Br J Radiol. 2026 Jan 12;99(1179):450–8. doi: 10.1093/bjr/tqag001 (PMC13016999; doi:10.1093/bjr/tqag001)
Supplement: tqag001_Supplementary_Data [file tqag001_supplementary_data.zip › BJR-D-25-00573-48-49.pdf]

Supplementary Table 1. Patient Characteristics

		Men				Women			
Age Group (y)	VAT Group	Mean Age (SD)	Mean BMI (SD)†	Mean VAT Area (SD) (cm^2)	Mean VAT HU (SD)	Mean Age (SD)	Mean BMI (SD)†	Mean VAT Area (SD) (cm^2)	Mean VAT HU (SD)
**All ages**	Lo Area/Lo HU	52.0 (17.9)	25.3 (3.5)	118.3 (66.4)	-91.7 (6.7)	51.4 (18.1)	24.3 (4.0)	54.6 (33.1)	-89.2 (6.2)
Men: N=65596	Lo Area/Hi HU	53.0 (18.2)	25.4 (4.3)	101.9 (67.0)	-78.9 (7.1)	51.9 (18.5)	24.2 (4.6)	48.4 (31.8)	-77.4 (5.8)
Women: N=71299	Hi Area/Lo HU	53.9 (16.3)	31.4 (5.2)	312.1 (110.0)	-101.4 (3.8)	52.8 (16.9)	32.8 (6.8)	194.0 (88.3)	-98.6 (4.5)
	Hi Area/Hi HU	54.4 (16.6)	33.8 (7.0)	308.3 (121.0)	-90.6 (6.2)	52.9 (17.3)	35.6 (8.9)	195.6 (108.6)	-87.3 (6.4)
**Age18-39**	Lo Area/Lo HU	26.8 (6.0)	23.6 (3.2)	46.1 (26.0	-88.8 (6.5)	27.5 (6.4)	23.4 (3.9)	25.3 (11.3)	-86.6 (5.7)
Men: N=15318	Lo Area/Hi HU	27.0 (6.3)	24.1 (3.7)	40.7 (23.2)	-77.1 (5.2)	27.5 (6.2)	23.5 (4.2)	23.7 (11.2)	-75.6 (4.4)
Women: N=18262	Hi Area/Lo HU	31.7 (5.4)	30.6 (5.4)	206.1 (84.9	-99.7 (4.1)	30.8 (5.9)	33.3 (7.8)	133.1 (77.9)	-95.5 (4.7)
	Hi Area/Hi HU	31.3 (5.7)	32.7 (7.5)	192.9 (95.5)	-89.8 (6.2)	30.6 (6.0)	34.5 (9.2)	122.4 (86.4)	-85.1 (5.8)
**Age 40-59**	Lo Area/Lo HU	49.4 (5.6)	25.8 (3.5)	121.6 (52.1)	-93.1 (6.4)	49.7 (5.5)	24.7 (4.1)	54.9 (27.4)	-90.1 (5.8)
Men: N=24694	Lo Area/Hi HU	50.7 (5.6)	25.9 (4.4)	104.2 (55.8)	-80.2 (7.6)	49.7 (5.6)	24.7 (4.5)	48.4 (26.2)	-78.4 (5.7)
Women: N=27588	Hi Area/Lo HU	50.7 (5.5)	32.1 (5.3)	318.1 (89.8)	-102.4 (3.3)	50.9 (5.4)	33.4 (6.6)	200.6 (79.5)	-99.8 (3.7)
	Hi Area/Hi HU	51.4 (5.4)	35.0 (7.4)	320.0 (105.3)	-91.4 (6.5)	50.6 (5.6)	36.8 (9.3)	208.8 (107.3)	-88.6 (6.3)
**Age 60-79**	Lo Area/Lo HU	67.2 (5.3)	25.8 (3.3)	159.1 (60.2)	-92.5 (6.4)	67.4 (5.5)	24.9 (4.3)	76.9 (34.0)	-91.0 (6.0)
Men: N=21723	Lo Area/Hi HU	68.4 (5.6)	25.9 (4.5)	136.7 (69.7)	-79.2 (7.5)	68.7 (5.7)	24.5 (4.9)	66.9 (36.1)	-78.4 (6.5)
Women: N=20890	Hi Area/Lo HU	67.9 (5.4)	31.5 (4.9)	371.9 (93.8)	-101.8 (3.7)	67.8 (5.4)	32.6 (6.2)	235.0 (81.3)	-100.0 (3.8)
	Hi Area/Hi HU	68.6 (5.5)	33.8 (6.2)	369.8 (101.2)	-90.7 (5.8)	68.4 (5.6)	36.0 (8.3)	240.3 (102.2)	-87.9 (6.5)
**Age 80-109**	Lo Area/Lo HU	84.3 (3.9)	24.5 (3.0)	154.5 (56.1)	-89.1 (7.1)	85.1 (4.1)	23.3 (3.7)	69.0 (29.1)	-86.5 (6.5)
Men: N=3861	Lo Area/Hi HU	85.1 (4.0)	24.5 (3.5)	133.8 (64.0)	-76.3 (6.8)	86.2 (4.5)	22.9 (3.8)	62.4 (31.4)	-74.2 (5.4)
Women: N=4559	Hi Area/Lo HU	84.0 (3.6)	29.2 (4.1)	358.0 (93.1)	-100.4 (4.4)	84.4 (3.8)	29.4 (5.0)	211.7 (76.7)	-97.7 (4.5)
	Hi Area/Hi HU	84.2 (3.6)	30.5 (4.7)	345.0 (87.9)	-89.1 (5.7)	85.2 (4.2)	30.4 (5.7)	202.0 (72.3)	-85.2 (6.3)

† 73% of men and 74% of women with VAT Area and HU measurements had a BMI measurement within 365 days of the date of their CT scan.
Lo, Low; Hi, High.

Supplementary Table 2. Thresholds for VAT Area quartiles and HU medians

		**Men**		**Women**	
Age Group (y)	VAT Area	VAT Area Quartile Thresholds (cm^2)	VAT HU Median	VAT Area Quartile Thresholds (cm^2)	VAT HU Median
**Age18-39**	Q1<br>Q2<br>Q3<br>Q4	39.6<br>94.3<br>178.4	-79.9<br>-85.0<br>-92.6<br>-98.4	23.3<br>47.6<br>104.7	-79.0<br>-82.5<br>-87.7<br>-94.0
**Age 40-59**	Q1<br>Q2<br>Q3<br>Q4	116.7<br>202.3<br>295.4	-81.9<br>-92.3<br>-97.4<br>-98.8	48.8<br>103.4<br>180.1	-80.9<br>-87.8<br>-93.9<br>-96.5
**Age 60-79**	Q1<br>Q2<br>Q3<br>Q4	154.9<br>249.3<br>349.3	-81.2<br>-91.4<br>-96.0<br>-98.0	71.8<br>134.2<br>213.7	-80.8<br>-89.3<br>-94.3<br>-95.9
**Age 80-109**	Q1<br>Q2<br>Q3<br>Q4	151.4<br>238.1<br>332.0	-77.3<br>-87.6<br>-93.3<br>-97.4	66.0<br>118.6<br>188.1	-76.7<br>-84.0<br>-90.5<br>-94.5
