# Supplementary figures and images for: Automated CT-based visceral fat density predicts mortality regardless of visceral fat area
Source: Br J Radiol. 2026 Jan 12;99(1179):450–8. doi: 10.1093/bjr/tqag001 (PMC13016999; doi:10.1093/bjr/tqag001)

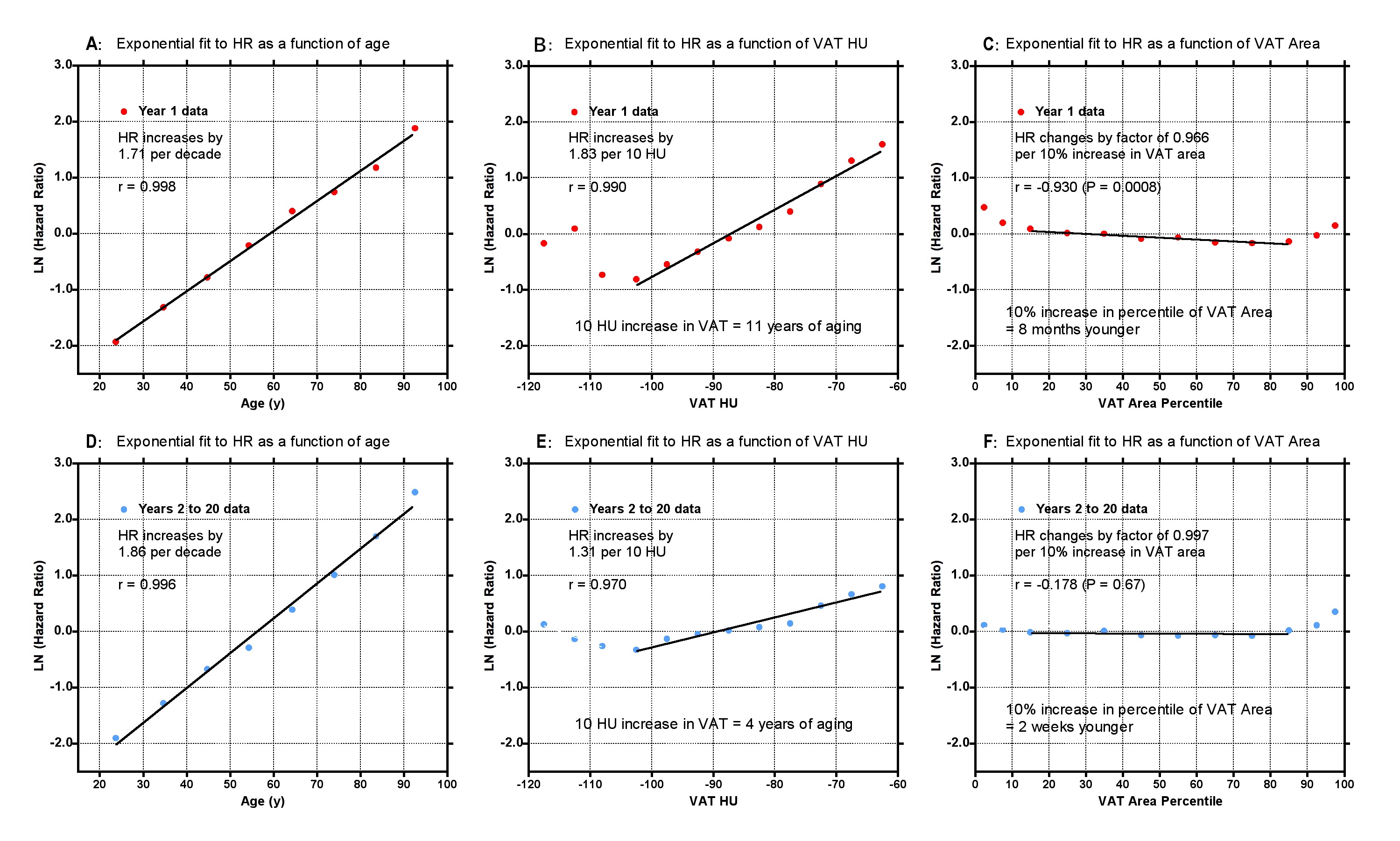

Supplement: tqag001_Supplementary_Data [file tqag001_supplementary_data.zip › BJR Figure S4.jpg]

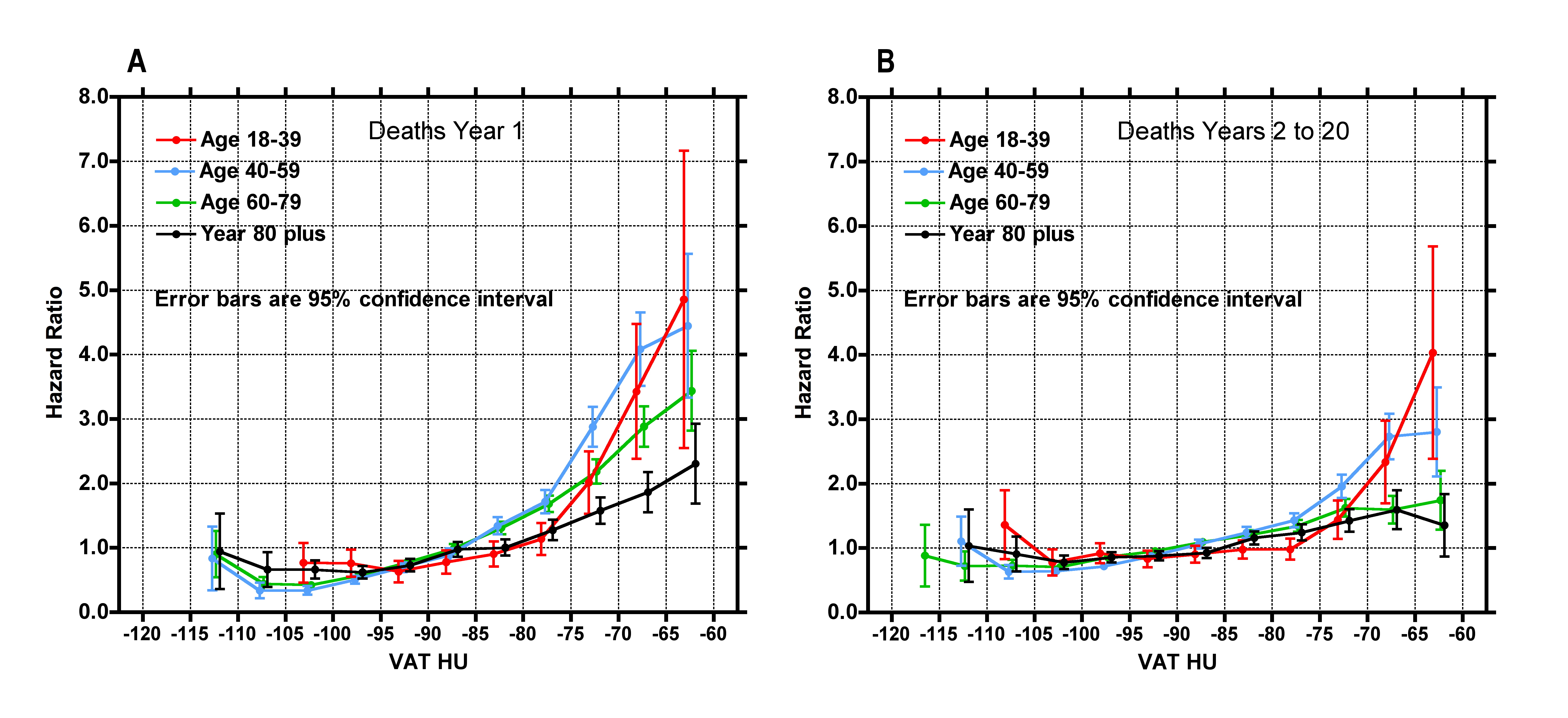

Supplement: tqag001_Supplementary_Data [file tqag001_supplementary_data.zip › BJR Revised Figure S1.jpg]

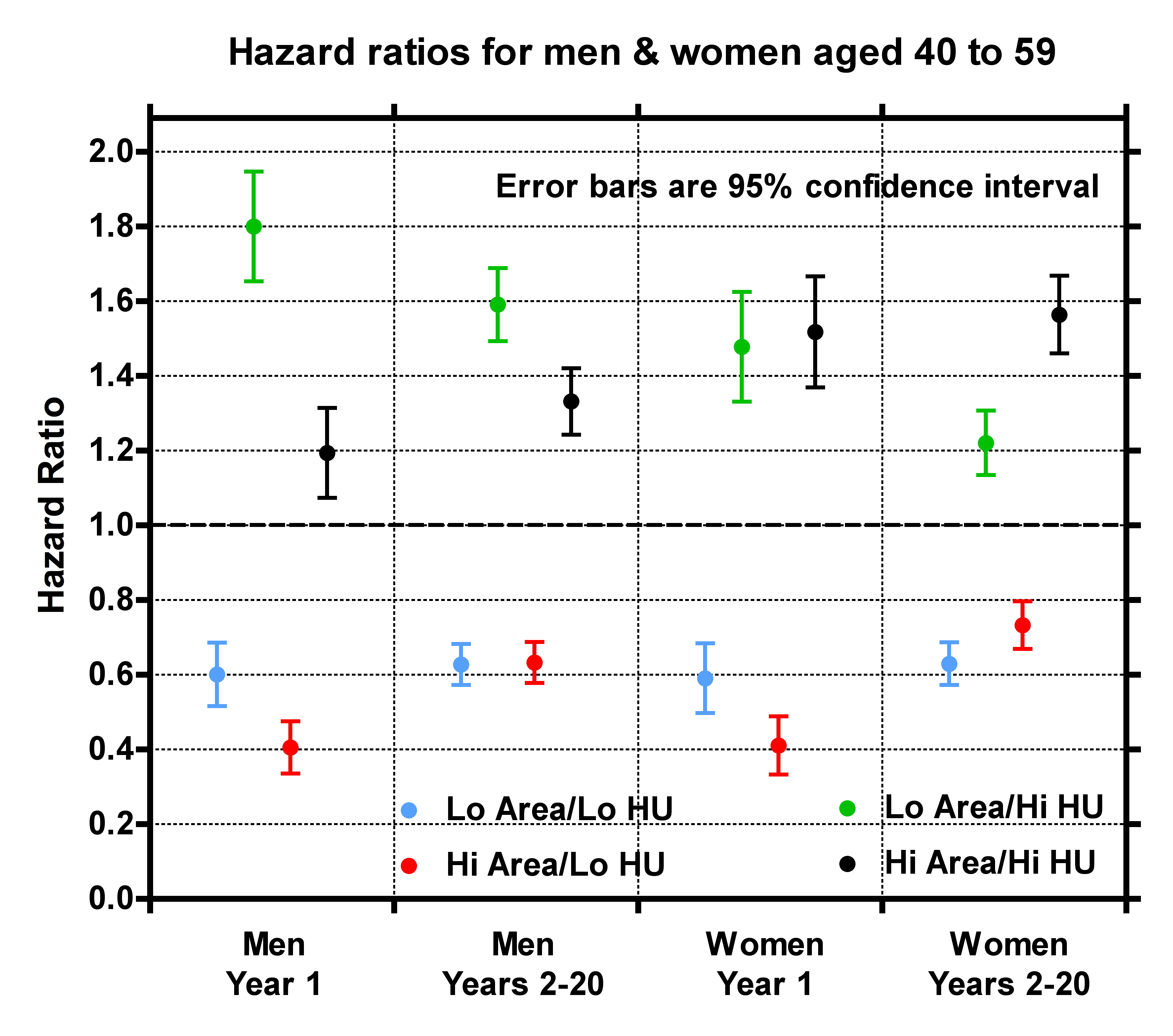

Supplement: tqag001_Supplementary_Data [file tqag001_supplementary_data.zip › BJR Revised Figure S2.jpg]

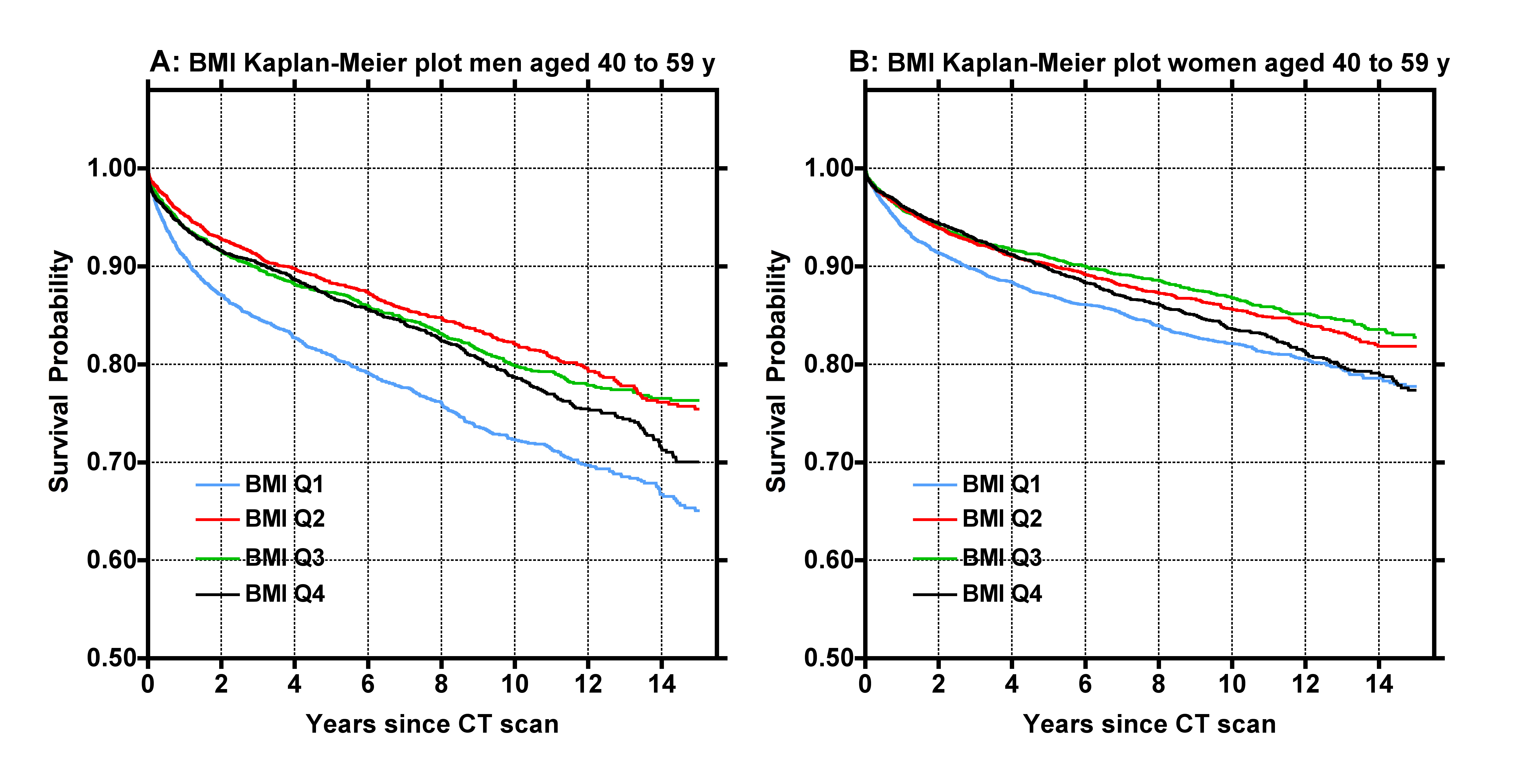

Supplement: tqag001_Supplementary_Data [file tqag001_supplementary_data.zip › BJR Revised Figure S3.jpg]
